# Supplementary material for: Adiposity Phenotypes and Associated Cardiometabolic Risk Profile in the Inuit Population of Nunavik
Source: Nutrients. 2024 Mar 2;16(5):725. doi: 10.3390/nu16050725 (PMC10934199; doi:10.3390/nu16050725)
Supplement: Supplementary file 1 [file nutrients-16-00725-s001.zip › nutrients-2870635-supplementary.pdf]

Supplementary Table S1. Associations between anthropometric indices and body fat composition measures among Inuit

	Weight				BMI				WC				WHtR			
	β-Coeff	$95\%_{CI}$		p-value	β-Coeff	$95\%_{CI}$		p-value	β-Coeff	$95\%_{CI}$		p-value	β-Coeff	$95\%_{CI}$		p-value
**Males**																
Percentage of body fat	0.44	0.41	0.47	<0.05	1.51	1.44	1.57	<0.05	0.51	0.48	0.54	<0.05	89.93	85.1	94.7	<0.05
Body fat mass	0.63	0.60	0.64	<0.05	2.02	1.97	2.09	<0.05	0.69	0.66	0.71	<0.05	116.90	111.32	122.51	<0.05
VAT level	0.27	0.26	0.28	<0.05	0.87	0.84	0.91	<0.05	0.30	0.28	0.31	<0.05	50.64	47.95	53.33	<0.05
**Females**																
Percentage of body fat	0.47	0.45	0.49	<0.05	1.33	1.29	1.38	<0.05	0.49	0.47	0.52	<0.05	77.36	73.56	81.17	<0.05
Body fat mass	0.72	0.70	0.73	<0.05	1.87	1.84	1.91	<0.05	0.67	0.65	0.70	<0.05	101.65	96.87	106.43	<0.05
VAT level	0.31	0.29	0.31	<0.05	0.81	0.78	0.84	<0.05	0.29	0.28	0.31	<0.05	45.67	43.32	48.02	<0.05

Values are β- Regression coefficient (95%CI) for 1 unit increase in weight, BMI, WC, WHR. All models are adjusted for age

Abbreviations: BMI, body mass index; WC, waist circumference; VAT level, visceral adipose tissue level; WHR, Waist-to-height ratio;

Supplementary Table S2. Association between anthropometric indices and cardiometabolic risk factors among Inuit

	**Weight**				**BMI**				**WC**				**WHtR**				**VFL**			
	β-Coeff	95%$_{CI}$		p-value	β-Coeff	95%$_{CI}$		p-value	β-Coeff	95%$_{CI}$		p-value	β-Coeff	95%$_{CI}$		p-value	β-Coeff	95%$_{CI}$		p-value
LDL-C	0.0095	0.0048	0.014	<0.05	0.027	0.013	0.042	<0.05	0.003	-0.0085	0.015	0.5	0.28	-1.56	2.12	0.8	0.04	0.0020	0.078	<0.05
HDL-C	-0.012	-0.013	-0.010	<0.05	-0.035	-0.039	-0.031	<0.05	-0.0015	-0.0049	0.0019	0.4	-0.078	-0.62	0.46	0.8	-0.015	-0.027	-0.0047	<0.05
TRIG	0.024	0.020025	0.028	<0.05	0.073	0.063	0.086	<0.05	0.012	0.0023	0.023	<0.05	2.28	0.69	3.87	<0.05	0.051	0.018	0.083	<0.05
CHOL/HDL	0.034	0.030	0.038	<0.05	0.099	0.089	.11	<0.05	0.012	0.0029	0.021	<0.05	1.43	-0.018	2.88	0.06	0.051	0.021	0.081	<0.05
APOB	0.013	0.011	0.015	<0.05	0.0043	0.0036	0.0052	<0.05	0.0023	0.00050	0.0041	<0.05	0.41	0.11	0.69	<0.05	0.0070	0.00099	0.013	<0.05
HBA1c [1]	0.0028	-0.0038	0.0094	0.4	0.0040	-0.016	0.024	0.7	0.0043	-0.012	0.021	0.6	-0.036	-2.60	2.51	0.8	-0.036	-0.058	0.070	0.6
Fasting glucose [1]	0.0088	0.0061	0.011	<0.05	0.028	0.019	0.036	<0.05	0.0053	-0.00048	0.011	0.07	0.97	0.031	1.91	<0.05	0.01	-0.011	0.031	0.4
HOMA-IR [1]	0.085	0.074	0.095	<0.05	0.24	0.21	0.27	<0.05	0.0017	-0.022	0.025	0.9	-2.86	-6.63	0.90	0.1	0.061	-0.025	0.15	0.2
SBP	0.32	0.28	0.36	<0.05	0.89	0.78	1.01	<0.05	0.014	-0.082	0.11	0.8	-4.87	-10.07	10.33	0.5	0.18	-0.14	0.49	0.3
DBP	0.17	0.15	0.21	<0.05	0.50	0.41	0.58	<0.05	0.060	-0.0069	0.13	0.08	5.15	-5.62	15.91	0.3	0.31	0.077	0.52	<0.05

Values are β- Regression coefficient (95%CI) for 1 unit increase in weight, BMI, WC, WHR or VFL. All models are adjusted for age, sex, smoking status and BMI in case of WC or VAT level

[1]Adjusted for age, sex, smoking status and T2D diagnosis

Abbreviations: BMI, body mass index; WC, waist circumference; VFL, visceral fat level; Waist-to-height ratio; ApoB, Alipoprotein-B; HDL-C, high-density lipoprotein; LDL-C, low-density lipoprotein; TG, triglycerides; HbA1c, Glycated hemoglobin

Supplementary Table S3. Subgroup analysis of the association between anthropometric indices and cardiometabolic risk factors in Inuit men

	Weight				BMI				WC				WHtR				VFL			
	β-Coeff	95% IC		p-value	β-Coeff	95% IC		P-value	β-Coeff	95% IC		p-value	β-Coeff	95% IC		p-value	β-Coeff	95% IC		p-value
LDL-C	0.009	0.0046	0.014	<0.05	0.03	0.016	0.047	<0.05	0.0035	-0.010	0.0170	0.6	0.22	-2.13	2.58	0.9	0.033	-0.010	.077	0.1
HDL-C	-0.009	-0.012	-0.0077	<0.05	-0.03	-0.040	-0.027	<0.05	-0.002	-0.0079	0.0038	0.1	-1.24	-2.68	0.19	0.09	-0.044	-0.0067	0.05	0.05
TG	0.029	0.020	0.039	<0.05	0.098	0.069	0.13	<0.05	0.0097	-0.016	0.036	0.1	3.57	-2.87	10.01	0.3	0.084	0.25	<0.05	<0.05
CHOL/HDL	0.039	0.032	0.048	<0.05	0.13	0.11	0.16	<0.05	0.0088	-0.013	0.031	0.8	2.66	-2.78	8.11	0.3	0.074	0.21	<0.05	<0.05
APOB	0.005	0.0039	0.0065	<0.05	0.018	0.013	0.022	<0.05	0.0020	-0.0016	0.0057	0.3	0.47	-0.42	1.36	0.3	0.000015	0.023	0.05	0.05
HbA1c[1]	0.0090	-0.0042	0.022	0.2	0.028	0.0016	0.071	0.2	0.0011	-0.036	0.038	0.5	1.16	-8.10	10.40	0.8	-0.11	0.13	1.0	1.0
Fasting glucose [1]	0.0070	0.0029	0.011	<0.05	0.024	0.011	0.038	<0.05	0.0053	-0.0051	0.016	0.7	2.63	-0.42	5.70	0.09	0.0038	0.078	<0.05	<0.05
HOMA-IR	0.084	0.065	0.10	<0.05	0.28	0.21	0.34	<0.05	0.018	-0.033	0.068	0.6	6.41	9.40	0.4	0.08	-0.11	-0.26	0.4	0.4
SBP	0.34	0.27	0.40	<0.05	1.10	0.90	1.30	<0.05	-0.077	-0.25	0.095	0.6	-28.08	-70.79	14.71	0.1	-0.43	0.69	0.65	0.65
DBP	0.21	0.16	0.25	<0.05	0.67	0.52	0.82	<0.05	0.068	-0.059	0.20	0.4	8.83	-22.77	40.43	0.5	0.067	0.89	<0.05	<0.05

Values are β- Regression coefficient (95%CI) for 1 unit increase in weight, BMI, WC or VAT level. All models are adjusted for age, smoking status and BMI in case of WC or VFL

[1]Adjusted for age, smoking status and T2D diagnosis

Abbreviations: BMI, body mass index; WC, waist circumference; VFL, visceral fat level; WHR, Waist-to-height ratio; ApoB, Alipoprotein-B; HDL-C, high-density lipoprotein; LDL-C, low-density lipoprotein; TG, triglycerides; HbA1c, Glycated hemoglobin

Supplementary Table S4. Subgroup analysis of the association between anthropometric indices and cardiometabolic risk factors in Inuit premenopausal women (estimated by age under 55 years old)

	Weight				BMI				WC				WHR				VFL			
	β-Coeff	$95\%_{IC}$		p-value	β-Coeff	$95\%_{IC}$		p-value	β-Coeff	$95\%_{IC}$		p-value	β-Coeff	$95\%_{IC}$		p-value	β-Coeff	$95\%_{IC}$		p-value
LDL-C	0.012	0.0044	0.021	<0.05	0.036	.011	.061	<0.05	0.0019	-0.017	0.021	0.8	0.19	-2.71	3.10	0.9	0.043	-0.022	0.11	0.2
HDL-C	-0.011	-0.014	-0.0095	<0.05	-0.031	-0.040	-0.028	<0.05	-0.00090	-0.0053	0.0035	0.7	-0.15	-0.81	0.52	0.7	-0.0097	-0.025	0.0052	0.2
TG	0.021	0.017	0.025	<0.05	0.065	0.054	0.076	<0.05	0.0068	-0.0023	0.016	0.1	1.96	0.59	3.34	<0.05	-0.0082	-0.039	0.023	0.6
CHOL/HDL	0.031	0.027	0.035	<0.05	0.091	0.079	0.10	<0.05	0.0070	-0.0023	0.016	0.1	1.41	-0.0084	2.83	0.05	0.0025	-0.029	0.034	0.9
APOB	0.0043	0.0034	0.0054	<0.05	0.013	0.010	0.016	<0.05	0.017	-0.00038	0.0040	0.1	0.40	0.071	0.72	<0.05	0.0035	-0.0038	0.011	0.3
HbA1c	0.0059	-0.0091	0.0079	0.9	-0.0063	-0.033	0.02	0.6	0.0020	-0.022	0.018	0.8	-0.84	-3.89	2.21	0.6	0.0048	-0.063	0.073	0.9
Fasting glucose	0.011	0.0072	0.014	<0.05	0.028	0.018	0.039	<0.05	0.00052	-0.0066	0.0076	0.9	-0.21	-1.31	0.90	0.7	-0.018	-0.046	0.011	0.2
HOMA-IR	0.088	0.071	0.10	<0.05	0.24	0.19	0.28	<0.05	-0.29	-0.056	-0.0011	<0.05	-7.29	-11.61	-2.98	<0.05	0.018	-0.094	0.13	0.7
SBP	0.36	0.30	0.41	<0.05	0.96	0.81	1.11	<0.05	0.042	-0.069	0.15	0.4	0.11	-0.27	0.48	0.6	0.90	-16.02	17.8	0.9
DBP	0.18	0.14	0.22	<0.05	0.50	0.39	0.62	<0.05	0.054	-0.033	0.14	0.2	7.61	-5.52	20.71	0.3	0.15	-0.14	0.45	0.3

Values are β- Regression coefficient (95%CI) for 1 unit increase in weight, BMI, WC or VAT level. All models are adjusted for age, smoking status and BMI in case of WC or VFL

[1]Adjusted for age, smoking status and T2D diagnosis

Abbreviations: BMI, body mass index; WC, waist circumference; Visceral fat level ; ApoB, Alipoprotein-B; HDL-C, high-density lipoprotein; LDL-C, low-density lipoprotein; TG, triglycerides; HbA1c, Glycated hemoglobin

Supplementary Table S5. Subgroup analysis of the association between anthropometric indices and cardiometabolic risk factors in Inuit aged 20 years old and more

	**Weight**				**BMI**				**WC**				**WHR**				**VFL**			
	β-Coeff	95%IC		p-value	β-Coeff	95%IC		p-value	β-Coeff	95%IC		p-value	β-Coeff	95%IC		p-value	β-Coeff	95%IC		p-value
LDL-C	0.0062	0.0022	0.01	<0.05	0.017	0.0057	0.028	<0.05	0.002	-0.0068	0.012	0.5	0.24	-1.21	1.69	0.7	0.03	0.0008	0.06	<0.05
HDL-C	-0.013	-0.014	-0.011	<0.05	-0.038	-0.043	-0.034	<0.05	-0.0013	-0.0049	0.0022	0.5	-0.061	-0.63	0.51	0.8	-0.017	-0.029	-0.0058	<0.05
TG	0.27	0.022	0.031	<0.05	0.080	0.065	0.094	<0.05	0.012	0.0017	0.024	<0.05	2.28	0.51	4.05	<0.05	0.065	0.027	0.10	<0.05
CHOL/HDL	0.036	0.031	0.040	<0.05	0.10	0.09	0.11	<0.05	0.012	0.0024	0.022	<0.05	1.47	-0.11	3.05	0.06	0.062	0.029	0.095	<0.05
APOB	0.004	0.0035	0.0052	<0.05	0.012	0.010	0.015	<0.05	0.0024	0.00041	0.0043	<0.05	0.42	0.12	0.73	<0.05	0.0078	0.0013	0.014	<0.05
HbA1c	0.0054	0.0028	0.0080	<0.05	0.022	0.014	0.029	<0.05	-0.0015	-0.0075	0.0045	0.6	0.43	-0.003	0.011	0.3	-0.0071	-0.0026	0.012	0.5
Fasting glucose	0.12	0.0084	0.016	<0.05	0.038	0.026	0.050	<0.05	0.0017	-0.0061	0.0097	0.7	0.91	-0.043	1.85	0.06	-0.0095	-0.038	0.019	0.5
HOMA-IR	0.088	0.077	0.097	<0.05	0.24	0.21	0.27	<0.05	-0.0013	-0.022	0.019	0.9	-3.42	-6.79	-0.047	<0.05	0.073	-0.0049	0.15	0.06
SBP	0.32	0.27	0.36	<0.05	0.90	0.77	1.037	<0.05	0.027	-0.076	0.13	0.6	-0.42	-16.81	15.95	0.9	0.25	-0.08	0.61	0.2
DBP	0.18	0.14	0.21	<0.05	0.51	0.41	0.60	<0.05	0.059	-0.013	0.13	0.1	7.13	-4.36	18.62	0.22	0.30	0.055	0.54	0.02

Values are β- Regression coefficient (95%CI) for 1 unit increase in weight, BMI, WC or VAT level. All models are adjusted for age, smoking status and BMI in case of WC or VFL

[1]Adjusted for age, smoking status and T2D diagnosis

Abbreviations: BMI, body mass index; WC, waist circumference; Visceral fat level ; ApoB, Alipoprotein-B; HDL-C, high-density lipoprotein; LDL-C, low-density lipoprotein; TG, triglycerides; HbA1c, Glycated hemoglobin

Supplementary Table S6. Ideal anthropometric indices cut-offs to detect cardiometabolic disorders among Inuit

	BMI kg/m^2			WC cm			WHtR			VLF		
	Cut-off	Sensitivity	Specificity	Cut-off	Sensitivity	Specificity	Cut-off	Sensitivity	Specificity	Cut-off	Sensitivity	Specificity
**Male**												
Diabetes	28.4	0.66	0.59	100	0.64	0.59	0.57	0.75	0.52	10	0.62	0.62
Prediabetes	28.8	0.62	0.60	100	0.62	0.60	0.63	0.72	0.53	10	0.61	0.64
Insulin resistance	26.7	0.46	0.72	93.5	0.46	0.72	0.58	0.53	0.66	8	0.43	0.76
Hypertension	25.6	0.52	0.68	86.5	0.52	0.68	0.51	0.62	0.63	7	0.48	0.71
Dyslipidemia	26.5	0.55	0.64	86.5	0.55	0.64	0.53	0.65	0.59	6	0.52	0.68
**Female**												
Diabetes	29.9	0.64	0.55	100	0.59	0.57	0.66	0.68	0.51	12	0.75	0.42
Prediabetes	26.6	0.62	0.56	93.5	0.58	0.58	0.62	0.70	0.53	13	0.73	0.43
Insulin resistance	24.6	0.49	0.70	92.0	0.47	0.69	0.58	0.53	0.68	14	0.61	0.57
Hypertension	28.9	0.54	0.59	92.5	0.52	0.60	0.61	0.60	0.56	13	0.68	0.46
Dyslipidemia	27.9	0.57	0.59	96.5	0.55	0.60	0.63	0.64	0.56	13	0.71	0.46

Abbreviations: BMI, body mass index; WC, waist circumference; WHtR, waist-to-height ratio; VFL, visceral fat level

Supplementary Table S7. Obesity features and associated cardiometabolic comorbidities of Nunavimmiut men and women aged 50 years old and more

		Men		Women		p-value
N		139		233		
Obesity	n (%)	44	31.65	67	28.8	<0.05
Severe Obesity	n (%)	8	5.8	16	6.8	0.8
Abdominal obesity	n (%)	89	64.0	175	75.1	<0.05
Dyslipidemia	n (%)	46	33.1	93	39.9	0.2
Insulin resistance	n (%)	109	78.4	181	77.7	0.9
Pre-diabetes	n (%)	24	16.2	53	22.7	0.2
Type 2 diabetes	n (%)	7	5.1	17	7.3	0.4
Hypertension	n (%)	94	67.6	89	38.2	<0.05
Non-alcoholic Fatty Liver Disease[2]	n (%)	5	3.6	5	2.2	0.4
Advanced stage of liver fibrosis [3]	n (%)	0	0	1	0.42	0.4
